# Loss of Disabled-2 Expression in Pancreatic Cancer Progression

**DOI:** 10.1038/s41598-019-43992-z

**Published:** 2019-05-17

**Authors:** Barbara A. Hocevar

**Affiliations:** Department of Environmental and Occupational Health, School of Public Health, Indiana University, Bloomington, IN USA

**Keywords:** Pancreatic cancer, Cell signalling

## Abstract

Pancreatic ductal adenocarcinoma (PDAC) is a highly aggressive cancer type characterized by rapid metastasis and resistance to chemotherapy, properties that are shared by cancer stem cells (CSCs). In pancreatic cancer, tumor cells which possess the properties of CSCs also phenotypically resemble cells that have undergone epithelial-to-mesenchymal transition or EMT. Disabled-2 (Dab2) is a multifunctional scaffold protein frequently downregulated in cancer that has been linked to the process of EMT. However, the role of Dab2 in pancreatic cancer development and progression remains unclear. Downregulation of Dab2 expression in pancreatic cancer cell lines was found to trigger induction of genes characteristic of EMT and the CSC phenotype, while overexpression of Dab2 in the Panc1 cell line blocked the process of TGFβ-stimulated EMT. In addition, selective inhibition of the TGFβRI/RII receptors was found to reverse genes altered by Dab2 downregulation. Dab2 mRNA expression was found to be decreased in PDAC tumor samples, as compared to levels observed in normal pancreatic tissue. Methylation of the Dab2 gene promoter was demonstrated in Stage I PDAC tumors and in the MiaPaCa2 cell line, suggesting that promoter methylation may silence Dab2 expression early in pancreatic cancer progression. These results suggest that Dab2 may function as a tumor suppressor in pancreatic cancer by modulation of the TGFβ-stimulated EMT and CSC phenotype.

## Introduction

Pancreatic cancer is the fourth leading cause of cancer deaths in the United States and one of the most lethal forms of cancer diagnosed. Despite advances in understanding the underlying mechanisms driving disease progression^1^, median survival time following diagnosis remains <6 months, with a five year survival rate of 6%^2^. The poor prognosis for pancreatic cancer can be attributed in part to the rapid metastasis of pancreatic cancer and its resistance to conventional chemotherapy. The properties which endow a tumor cell with the ability to metastasize are similar to those possessed by normal stem cells, including self-renewal, migratory capability and drug-effluxing ability, leading to the theory that cancer arises from a putative cancer stem cell (CSC)^3^. Since pancreatic cancer recapitulates many of these same characteristics, it has also been postulated that it too originates from a stem cell or stem cell-like precursor cell^4^. CSCs, including those isolated from pancreatic cancer tumors, have been shown to express genes characteristic of a cell which has undergone epithelial-to-mesenchymal transition or EMT^5^.

EMT is characterized by a morphological change from an epithelial to a mesenchymal phenotype, with concomitant changes in gene expression from epithelial, such as E-cadherin, to mesenchymal, such as vimentin, fibronectin, and N-cadherin^6^. While EMT occurs normally in embryonic development, these same changes occur during tumor progression and metastasis^7^. EMT has been observed in resected pancreatic tumors and was associated with poor survival^8^. In addition, low E-cadherin expression, a hallmark for cells that have undergone EMT, was shown to be associated with dedifferentiation, advanced tumor stage and high incidence of lymph node metastasis in invasive pancreatic cancer^9–11^. Loss of E-cadherin expression during EMT is due to transcriptional repression, primarily mediated by the zinc-finger proteins Zeb1 and Zeb2 and the bHLH proteins Snail, Slug and Twist, whose increased expression in pancreatic cancer has been correlated with poor prognosis^11^. EMT has also been associated with drug resistance in pancreatic cancer^12,13^.

While activation of multiple signaling pathways such as Wnt, Notch and Hedgehog have been shown to trigger EMT, the TGFβ signaling pathway is believed to be a primary inducer of EMT in pancreatic cancer^14^. Increased TGFβ expression has been demonstrated in pancreatic cancer and was associated with poor prognosis^15,16^. TGFβ signaling is initiated following ligand binding to the TGFβRII/TGFβRI receptor complex, which results in engagement of downstream signaling molecules, including Smad2/3 and Smad4. Mutations or deletions of *SMAD4* are observed in 50% of pancreatic ductal adenocarcinoma (PDAC) cases^1^ and its deletion in the background of oncogenic Kras^G12D^ expression in mice has been shown to promote pancreatic tumor development and metastasis^17^. Activation of downstream TGFβ signaling is also controlled by TGFβ receptor recycling, which occurs through the endocytic pathway^18,19^. The adaptor protein Disabled-2 (Dab2) has been shown to play an important role in TGFβ receptor trafficking through the endosomal pathway^20^ thus participating in downstream TGFβ signaling^21^.

Dab2 is a scaffold protein that acts as a cargo-specific mediator of clathrin-mediated endocytosis as well as a regulator of multiple signaling pathways. First described as a transcript consistently downregulated in ovarian carcinoma^22^, the expression of Dab2 has been shown to be decreased in many cancer types including breast^23^, colorectal^24^, lung^25^, urothelial bladder^26^ and squamous cell carcinoma (SCC)^27^. We have previously shown that knockdown of Dab2 expression in normal human mammary epithelial cells led to upregulation of TGFβ2 expression and EMT^28^. Loss of Dab2 expression has been shown to be mediated by promoter methylation in SCC^27^, lung^29^, nasopharyngeal^30^ and hepatocellular carcinoma^31^ and through histone modification in transitional cell carcinoma^32^.

A previous study in pancreatic cancer has shown that primary tumor samples exhibited upregulated Dab2 expression, with decreased Dab2 expression observed in lymph node metastases and metastatic pancreatic cell lines, suggesting that Dab2 acted as an inhibitor of late tumor progression and metastasis^33^. The goal of this study was to investigate the expression of Dab2 during different stages of pancreatic cancer and to investigate if loss of Dab2 expression correlates with EMT and/or the CSC phenotype. Downregulation of Dab2 in pancreatic cancer cell lines led to altered gene expression patterns indicative of EMT and upregulation of cancer stem cell-specific markers. In addition, decreased Dab2 mRNA expression was shown in early stage pancreatic cancer where loss of Dab2 expression may be mediated in part by promoter methylation.

## Results

### Decreased Dab2 expression is correlated with an EMT phenotype in pancreatic cancer cell lines

**Figure 1:**
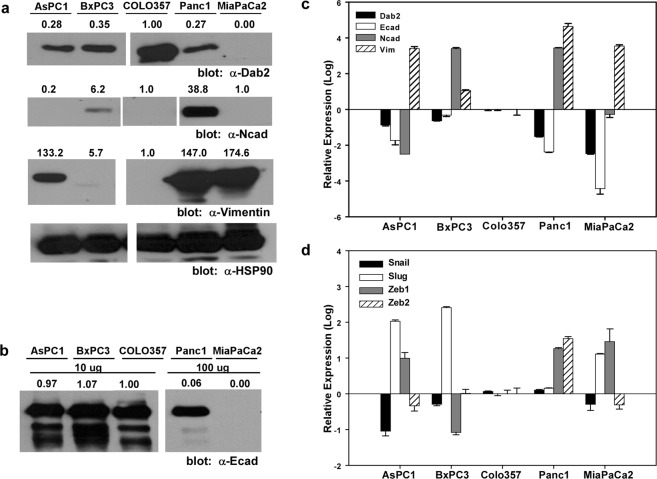
Dab2 levels correlate with EMT in pancreatic cancer cell lines. (**a**) Cell lysates were prepared from the indicated cell lines followed by Western blot analysis for Dab2, N-cadherin and vimentin as described in Methods. Equal protein loading is demonstrated by Western blot analysis for HSP90. Shown is a representative Western blot (n = 3 independent experiments). White space indicates removal of intervening lanes. Original blots can be found in Fig. S2. (**b**) E-cadherin expression is shown by Western blot analysis. A total of 10 μg of protein was used for detection in AsPC1, BxPC3 and COLO357 cells, while 100 μg of protein was used to demonstrate expression in Panc1 and MiaPaCa2 cells. Numbers above individual protein blots represent normalized expression to HSP90, with levels observed in COLO357 cells set to 1.0. White space indicates removal of intervening lanes. Original blots can be found in Fig. S2. (**c**,**d**) RNA expression of Dab2 and the indicated EMT marker proteins was measured by qRT-PCR analysis. Relative log expression levels are shown as compared to levels in COLO357 cells. Shown is the mean log expression value ± SD from an individual experiment; n = 3 independent experiments.

To assess whether the expression of Dab2 is linked to a specific mutational profile, mRNA and protein levels were determined in a panel of pancreatic cancer cell lines whose mutational status of *KRAS*, *INK4A*, *TP53*, and *SMAD4* has been previously determined (Table S1)^34–36^. Pancreatic cancer cell lines that express wild-type K-Ras, the COLO357 and BxPC3 cell lines, were found to exhibit higher protein and mRNA levels of Dab2 compared to AsPC1, Panc1 and MiaPaCa2 cell lines, which express a mutant form of the K-Ras protein (Fig. 1a,c). To assess whether decreased Dab2 expression is associated with an EMT phenotype in these cell lines, mRNA and protein expression of E-cadherin, N-cadherin and vimentin was determined (Fig. 1a–c). While calculation of the Pearson product-moment correlation coefficient demonstrated a strong positive correlation between Dab2 and E-cadherin mRNA expression (r = 0.975, n = 5, p = 0.005), this was not evident at the protein expression level. Vimentin expression was found to be highest in cell lines with low Dab2 expression, while N-cadherin expression did not correlate with Dab2 expression in these cell lines (Fig. 1b,c). The expression of the known transcriptional E-cadherin repressors Snail, Slug, Zeb1 and Zeb2 was also determined (Fig. 1d). While a clear correlation of mRNA expression of Snail, Slug and Zeb2 with Dab2 or E-cadherin expression levels was not consistent across cell lines, expression of Zeb1 was highest in the cell lines with low Dab2 and E-cadherin mRNA expression that also harbor mutant K-Ras (Fig. 1d).

### Dab2 expression affects EMT and CSC markers

**Figure 2:**
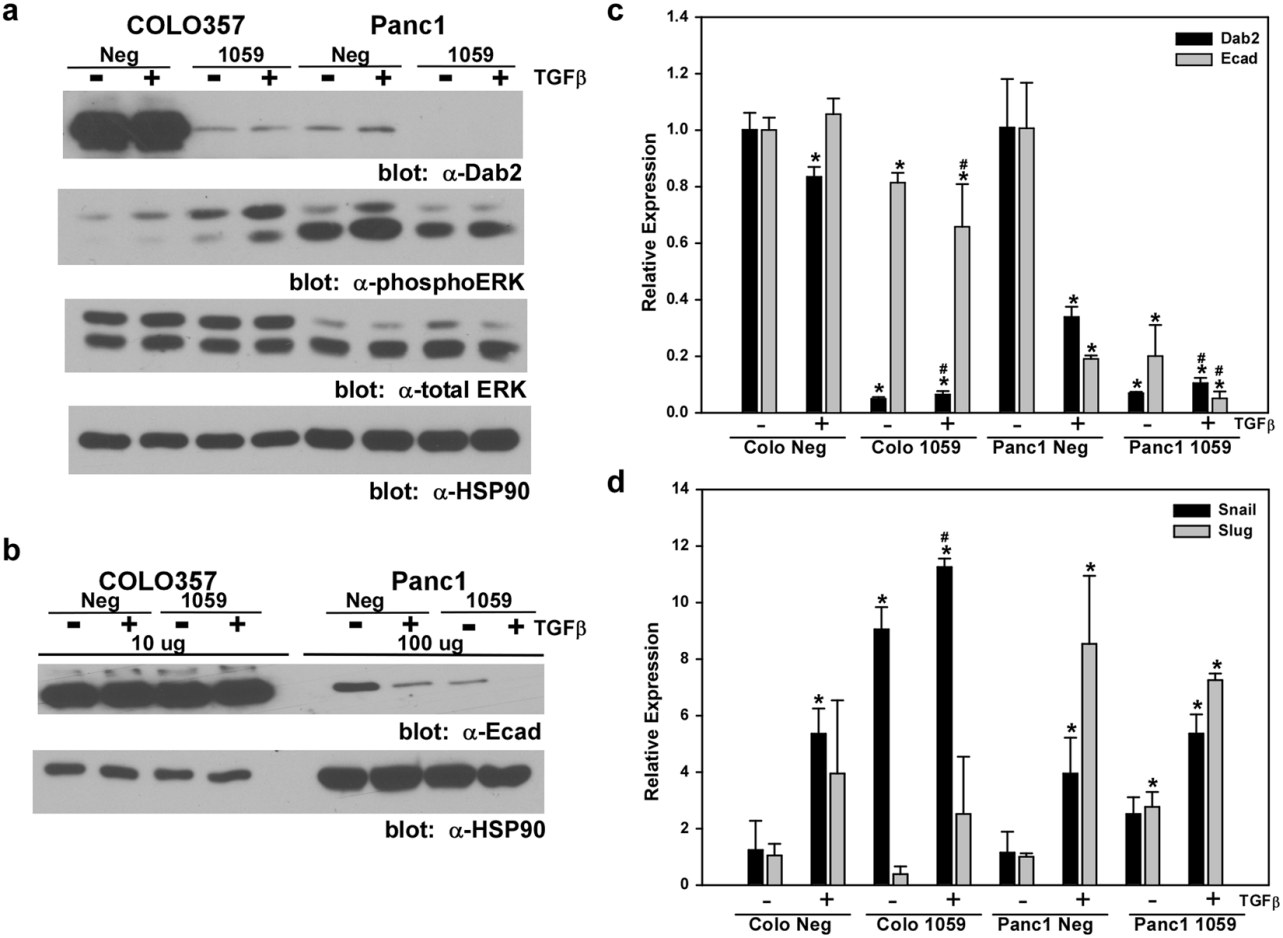
Stable knockdown of Dab2 stimulates EMT. (**a**,**b**) Cell lysates were prepared from untreated COLO357 Neg, COLO357 1059, Panc1 Neg and Panc1 1059 cells or cells treated with 2.5 ng/mL TGFβ1 for 72 hrs. Lysates were analyzed for protein expression of Dab2, phospho-ERK1/2, total ERK2, E-cadherin and HSP90 by Western blot analysis as detailed in the Methods section. For analysis of E-cadherin expression, 10 μg of protein was loaded in COLO357 Neg and COLO357 1059 cells, while 100 μg of protein was loaded from Panc1 Neg and Panc1 1059 cells. Western blot analysis for HSP90 was used to demonstrate equal protein loading. Shown is a representative Western blot (n = 3 independent experiments). Original blots can be found in Fig. S3. (**c**,**d**) mRNA expression for Dab2, E-cadherin, Snail and Slug was determined by qRT-PCR analysis in untreated COLO357 Neg, COLO357 1059, Panc1 Neg and Panc1 1059 cells or cells treated with 2.5 ng/mL TGFβ1 for 72 hrs. Relative expression is shown, with mRNA levels in the COLO357 Neg and Panc1 Neg cell lines for each gene set to 1.0 to allow comparison to their corresponding knockdown cell lines. Shown is the mean relative expression level ± SD performed in triplicate. * signifies p < 0.05 difference in expression for comparison to untreated Neg cell line; ^#^ represents p < 0.05 difference in expression for comparison to TGFβ1-treated Neg cell line.

To reflect the mutational heterogeneity present in PDAC, stable knockdown of Dab2 expression was established in COLO357 cells that express wild-type K-Ras and p53, and homozygous deletion of *SMAD4*, and in Panc1 cells that express mutant K-Ras, mutant p53, and wild-type Smad4 protein^34,36^. To induce EMT, cells were treated with TGFβ for 72 hrs. Introduction of the Dab2-targeted shRNA (1059) construct into the parental cell lines efficiently decreased both mRNA and protein levels of Dab2 (Fig. 2a,c). While TGFβ treatment of Panc1 Neg cells led to decreased E-cadherin protein and mRNA expression, TGFβ had no effect on E-cadherin levels in COLO357 Neg cells (Fig. 2b,c). Knockdown of Dab2 expression in Panc1 1059 cells led to decreased basal protein and mRNA expression of E-cadherin, which was further decreased with TGFβ treatment (Fig. 2b,c). In COLO357 1059 cells, decreased Dab2 expression also led to a decrease in E-cadherin mRNA levels; however no effect was seen at the protein level. To further characterize the transcriptional regulation of the E-cadherin gene in these cell lines, mRNA expression of known transcriptional repressors was determined (Fig. 2d). TGFβ treatment of Panc1 Neg cells increased Snail and Slug mRNA, while only Snail mRNA expression was increased in COLO357 Neg cells (Fig. 2d). Loss of Dab2 expression in Panc1 1059 cells resulted in increased basal expression of both Snail and Slug, which was not significantly altered by TGFβ treatment, while COLO357 1059 cells exhibited increased basal and TGFβ-stimulated expression of Snail mRNA levels (Fig. 2d). Analysis of basal and TGFβ-stimulated Zeb1 and Zeb2 mRNA levels in Dab2 knockdown cell lines compared to their respective control cell lines revealed no significant difference in expression for either the Panc1 or COLO357 cells (data not shown).

Loss of Dab2 expression has previously been shown to lead to activation of the MAPK pathway^28,37^, and MAPK activation has been shown to cooperate with TGFβ in induction and maintenance of stable EMT^38^. Loss of Dab2 in the COLO357 1059 cell line led to increased basal levels of p-ERK1/2, which was further augmented by TGFβ (Fig. 2a). In contrast, Panc1 Neg cells displayed elevated basal p-ERK1/2 levels, consistent with mutant K-Ras expression; however, loss of Dab2 in Panc1 1059 cells did not alter basal or TGFβ-stimulated p-ERK1/2 levels (Fig. 2a).

**Figure 3:**
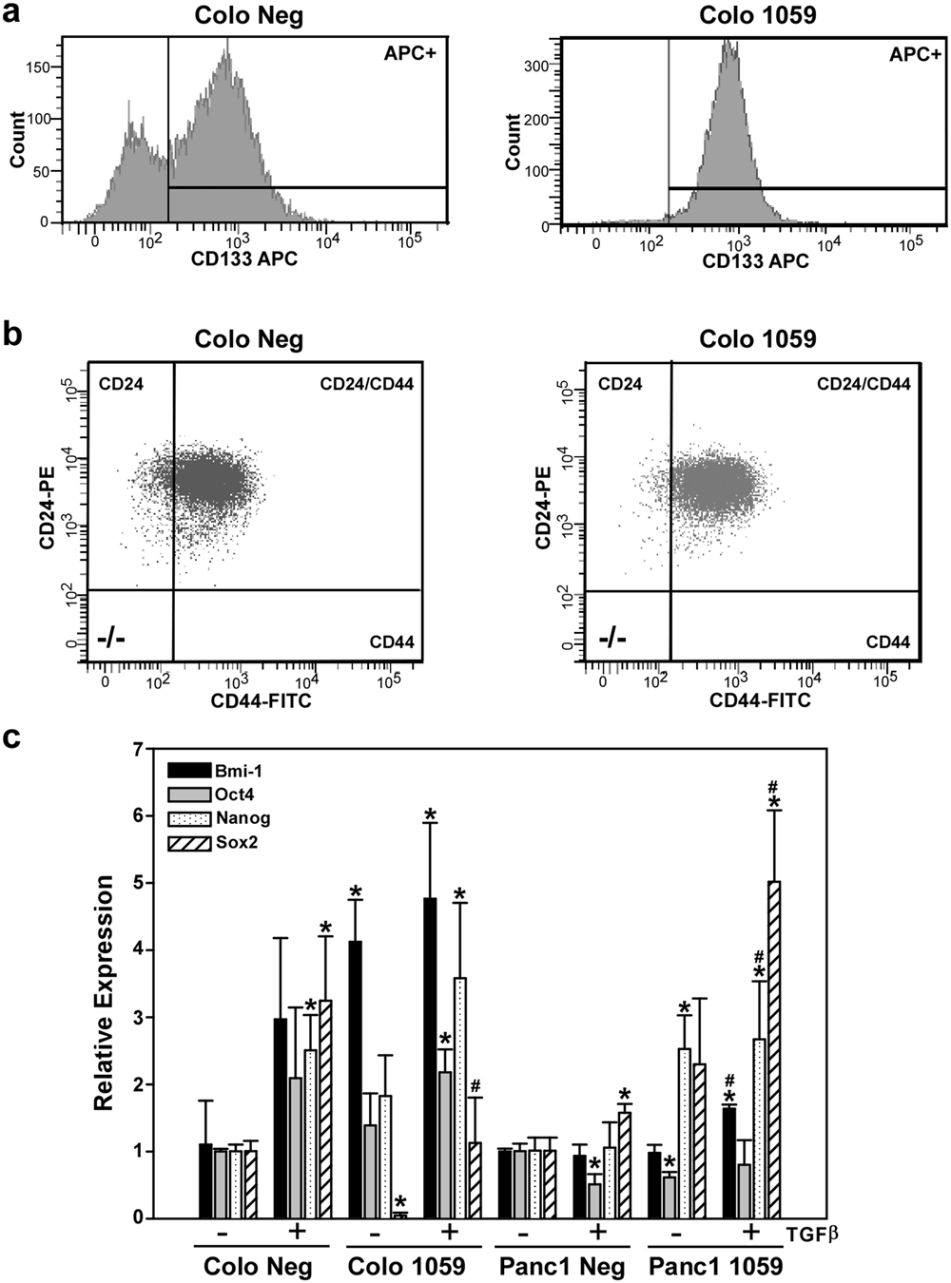
Decreased Dab2 expression leads to altered expression of CSC markers. (**a**,**b**) Cell surface expression of CD133, CD24 and CD44 was determined by flow cytometry in COLO 357 and COLO357 1059 cells as described in the Methods section. Shown is a representative histogram depicting expression levels. Numerical values of expression are provided in Table 1. (**c**) mRNA expression for Bmi-1, Oct4, Nanog and Sox2 was determined by qRT-PCR analysis in untreated COLO357 Neg, COLO357 1059, Panc1 Neg and Panc1 1059 cells or cells treated with 2.5 ng/mL TGFβ1 for 72 hrs. Relative expression is shown, with mRNA levels in the COLO357 Neg and Panc1 Neg cell lines for each gene set to 1.0 to allow comparison to their corresponding knockdown cell lines. Shown is the mean relative expression level ± SD performed in triplicate. * signifies p < 0.05 difference in expression for comparison to untreated Neg cell line; ^#^ represents p < 0.05 difference in expression for comparison to TGFβ1-treated Neg cell line.

In pancreatic cancer, cells that express the cell-surface proteins CD133^39^ or the combination of CD44/CD24/EpCAM^40^ have been shown to exhibit characteristics of stem cells. Analysis of putative stem cell markers by flow cytometry was performed to assess whether a decrease in expression of Dab2 alters their cell surface expression. Downregulation of Dab2 in the COLO357 cell line increased the cell surface expression of CD133 from 73% in the parental cell line to nearly 100% (Fig. 3a, Table 1). In contrast, expression of CD133 was low in the Panc1 cell line and was not significantly altered by loss of expression of Dab2 (Table 1). While the percentage of cells that concurrently express both CD24 and CD44 (CD24+/CD44+) or express cell-surface EpCAM was increased in both the COLO357 1059 and Panc1 1059 cell lines versus their respective control cells, the difference was not significant (Fig. 3b, Table 1).

**Table 1:** Cell Surface Expression of Stem Cell Markers (ave % ± SD).

Cell line	CD133+	CD24−/CD44−	CD24+/CD44−	CD24−/CD44+	CD24+/CD44+	EPCAM+
COLO357 Neg	72.9 ± 0.6	0.0 ± 0.0	11.1 ± 4.9	0.0 ± 0.0	88.9 ± 4.9	89.6 ± 0.4
COLO357 1059	98.4 ± 0.2*	0.0 ± 0.0	4.0 ± 2.5	0.0 ± 0.0	96.1 ± 2.5	91.0 ± 11.3
Panc1 Neg	0.4 ± 0.1	0.2 ± 0.2	0.0 ± 0.0	82.9 ± 5.7	16.8 ± 5.6	2.9 ± 0.4
Panc1 1059	0.8 ± 0.1	0.2 ± 0.1	0.0 ± 0.0	80.7 ± 9.2	19.2 ± 9.1	4.1 ± 1.0

*p < 0.05.

Expression of Bmi-1, Nanog, Oct4 and Sox2 has been implicated in maintenance of the stem cell phenotype in pancreatic cancer^41,42^. As shown in Fig. 3c, TGFβ treatment induced expression of Bmi-1, Oct4, Nanog and Sox2 in both COLO357 Neg and COLO357 1059 cell lines. While Dab2 downregulation in COLO357 1059 cells led to an increase in basal and TGFβ-stimulated expression of Bmi-1, Panc1 1059 cells showed increased basal and TGFβ-stimulated expression of Nanog and Sox2 (Fig. 3c). Together, these results suggest that downregulation of Dab2 may trigger an EMT phenotype concomitant with increased expression of cell surface markers and pluripotency factors that are indicative of the cancer stem cell phenotype.

**Figure 4:**
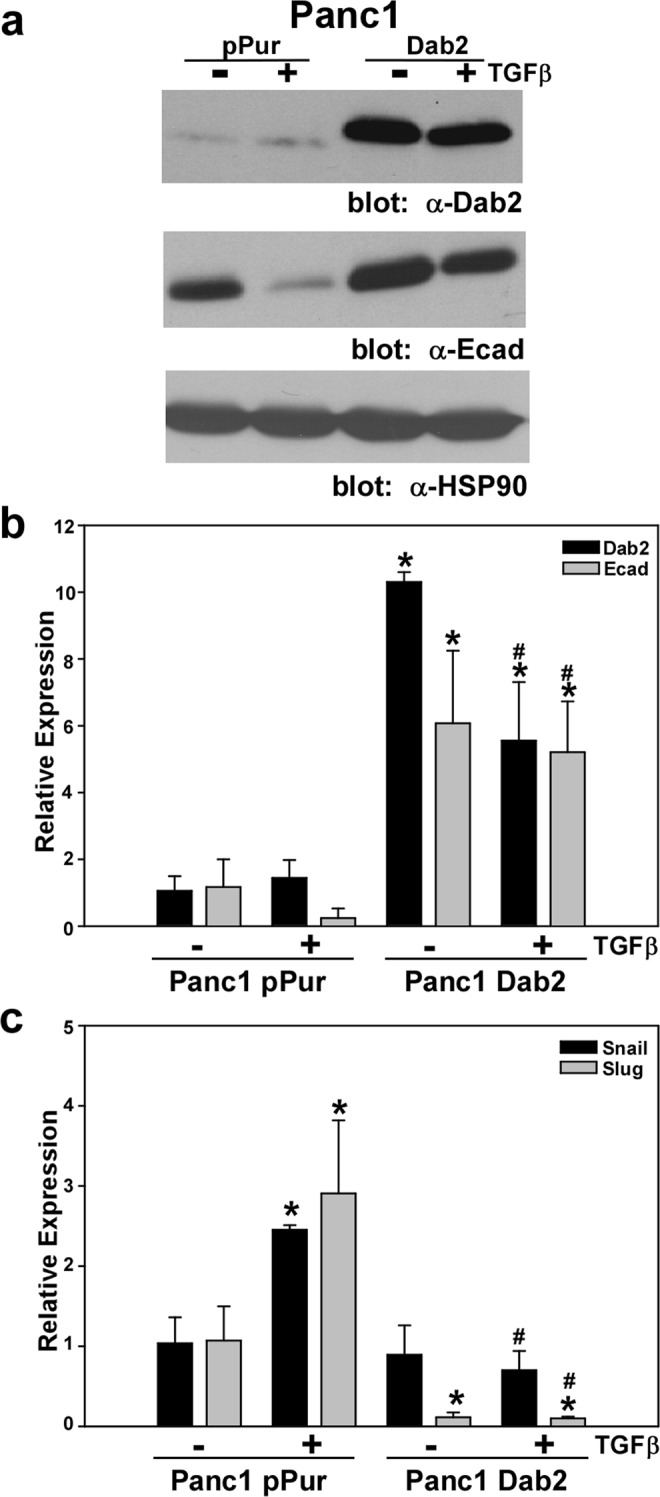
Overexpression of Dab2 prevents TGFβ-stimulated EMT. (**a**) Western blot analysis for Dab2, E-cadherin and HSP90 protein expression was performed on lysates prepared from Panc1pPur and Panc1Dab2 cell lines untreated or treated with TGFβ1 for 72 hrs. Analysis for HSP90 protein expression is used to show equal loading. Shown is a representative Western blot (n = 3 independent experiments). Original blots can be found in Fig. S4. (**b**,**c**) mRNA expression for Dab2, E-cadherin, Snail and Slug was determined by qRT-PCR analysis in untreated Panc1pPur and Panc1Dab2 cells or cells treated with 2.5 ng/mL TGFβ1 for 72 hrs. Relative expression is shown, with mRNA levels in the Panc1pPur cell line for each gene set to 1.0. Shown is the mean relative expression level ± SD performed in triplicate. * signifies p < 0.05 difference in expression for comparison to the untreated Panc1pPur cell line; ^#^ represents p < 0.05 difference in expression for comparison to TGFβ1-treated Panc1pPur cell line.

To assess the effect of restoration of Dab2 expression on TGFβ-stimulated EMT, Dab2 was overexpressed in the Panc1 cell line. Expression of Dab2 in Panc1 cells triggered ~8-fold higher basal E-cadherin mRNA and protein levels which was relatively unaffected by TGFβ treatment as compared to the Panc1 vector control cell line (Fig. 4a,b). While TGFβ treatment led to upregulation of Snail and Slug mRNA levels in Panc1 vector control cells, overexpression of Dab2 blocked this effect (Fig. 4c). These results suggested that Dab2 may help to maintain the epithelial phenotype by alteration of the TGFβ signaling pathway in pancreatic cancer cells. To address this mechanism, both the COLO 357- and Panc1-derived cell lines were treated with the selective TβRI/RII inhibitor LY2109761^43^. While LY2109761 treatment did not alter expression of all EMT or CSC genes, E-cadherin mRNA levels were increased while vimentin levels were decreased by LY2109761 in Panc1 1059 cells (Fig. S1a). In addition, mRNA expression of CD133 was decreased by LY2109761 in both COLO 357 Neg and 1059 cell lines (Fig. S1b).

### Loss of Dab2 expression occurs early in pancreatic cancer progression

**Figure 5:**
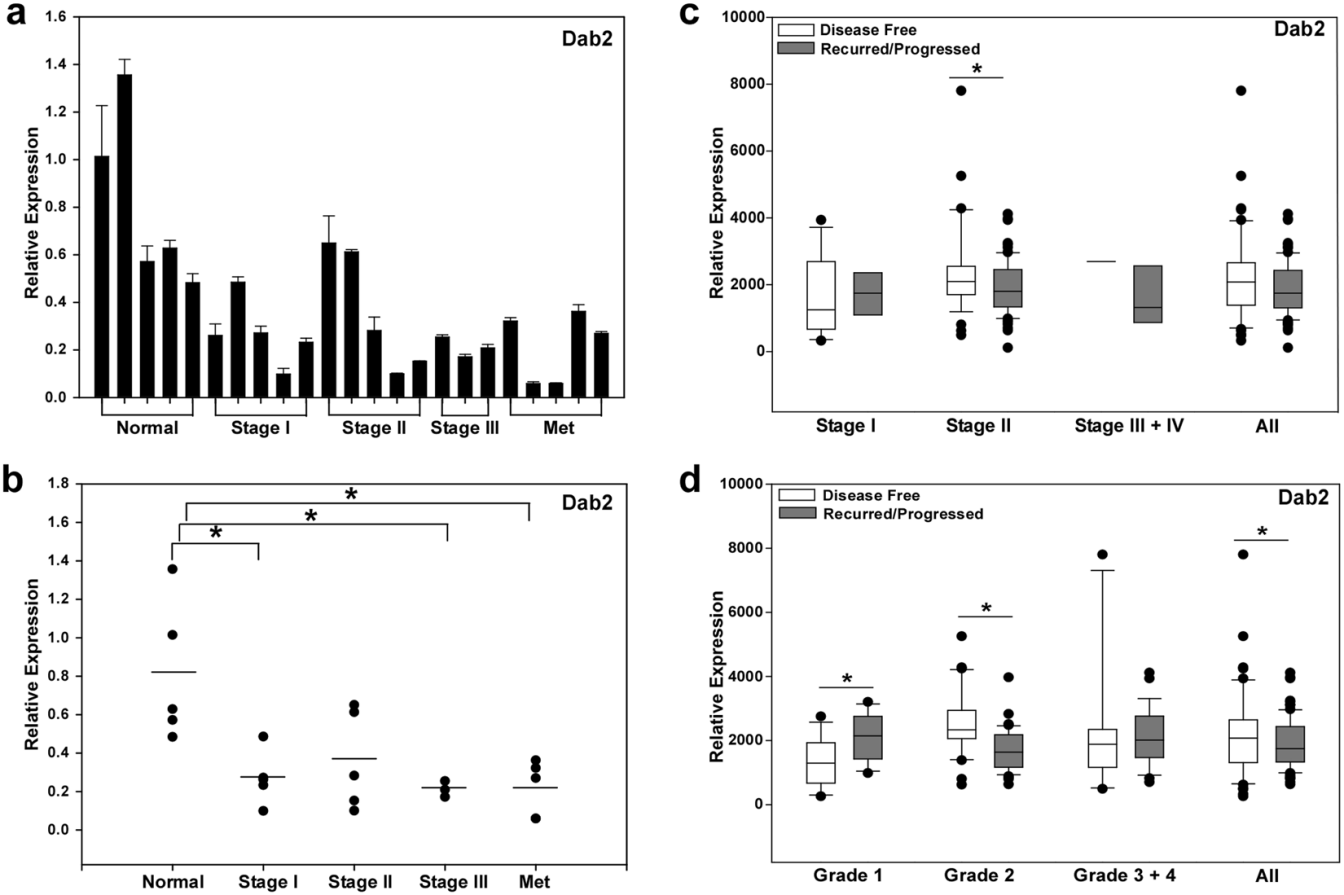
Decreased expression of Dab2 and E-cadherin expression in pancreatic cancer. (**a**) Dab2 mRNA expression was determined in individual normal and pancreatic tumor samples by qRT-PCR analysis as described in Methods. Shown is the mean value ± SD relative to the mean expression in normal samples, which was set to 1.0. (**b**) Stage-specific mRNA expression for Dab2 is shown for normal and pancreatic cancer tumor samples depicted in a. Mean expression values for each group is depicted by the bar. *p < 0.05. (**c**,**d**) mRNA Dab2 expression (RNA Seq V2 RSEM) and disease-free and recurred/progressed status was retrieved from the TCGA pancreatic adenocarcinoma dataset using cBioPortal. Shown is the box/whisker plots obtained from all tumors stratified by Stage (**c**) or Grade (**d**). * signifies p < 0.05.

To obtain a quantitative measurement of Dab2 expression at different stages of pancreatic ductal adenocarcinoma, qRT-PCR analysis was performed on mRNA isolated from normal pancreatic tissue (n = 5) and pancreatic tumor samples (n = 18). Normal pancreatic tissue samples used in the study were designated as normal adjacent tissue obtained from various pancreatic pathologies. The clinicopathological parameters of the normal pancreatic tissue and pancreatic tumor samples utilized is provided in Supplemental Data Table 2 (Table S2). Dab2 mRNA levels were found to be highest in normal pancreatic tissue, while mean Dab2 expression levels were significantly decreased in pancreatic cancer stages I, III, and metastatic tumor samples (p < 0.05; Fig. 5a,b). To extend these findings, mRNA expression of Dab2 in PDAC was interrogated in the TCGA database using the cBio Cancer Genomics Portal^44,45^. While overall Dab2 mRNA expression levels were not significantly different when stratified by stage, Dab2 mRNA levels were higher in individuals determined disease-free versus those designated with recurred/progressed disease in Stage II PDAC (Fig. 5c). When stratified by tumor grade, Dab2 levels were found to be significantly higher in disease-free versus recurred/progressed disease status, with Grade 2 tumors attaining the highest significance (Fig. 5d). We were unable to interrogate the difference in mRNA Dab2 levels between normal pancreatic tissue and the various stages or grades of pancreatic cancer, as normal pancreatic tissue was not included in this database.

**Figure 6:**
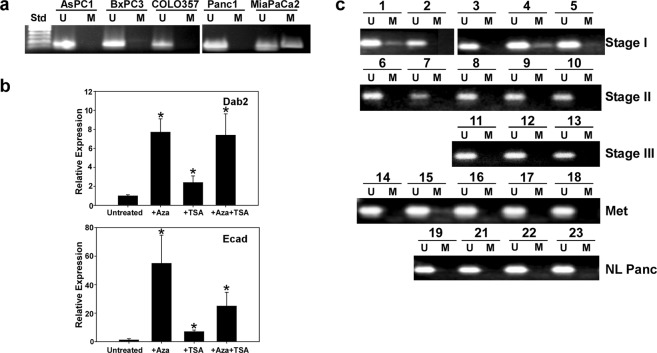
Dab2 promoter methylation in pancreatic tumor samples and pancreatic cancer cell lines. (**a**,**c**) Genomic DNA was isolated from the indicated pancreatic cancer cell lines and the normal or tumor pancreatic tissue samples as described under Methods. Following bisulfite modification of the DNA, methylation-specific PCR was performed using primers corresponding to unmethylated (U) and methylated (M) sequences derived from exon 2 of the Dab2 gene promoter. PCR products were separated on a 2% agarose gel and visualized by EtBr staining. (**b**) MiaPaCa2 cells were untreated or treated with 5-aza-2′deoxycytidine (Aza), trichostatin A (TSA) or the combination (Aza + TSA) as described in Methods. Following treatment, mRNA was isolated and qRT-PCR was performed to determine mRNA expression of Dab2 and E-cadherin. Relative expression is shown, with mRNA levels in untreated MiaPaCa2 cells set to 1.0. Shown is the mean relative expression level ± SD performed in triplicate. * signifies p < 0.05.

To determine whether loss of Dab2 expression in pancreatic cancer was due to promoter methylation, methylation-specific PCR analysis of the exon 1 promoter region of the Dab2 gene was performed on genomic DNA isolated from pancreatic cancer cell lines and normal pancreatic tissue and pancreatic tumor samples. Significant methylation of the Dab2 promoter in cell lines was observed only in the MiaPaCa2 cell line (Fig. 6a), which also exhibited the lowest mRNA level of Dab2 (Fig. 1c). Treatment with the DNA methyltransferase inhibitor, 5-azacytidine, resulted in an 8-fold upregulation of Dab2 mRNA levels in the MiaPaCa2 cells (Fig. 6b). Treatment of MiaPaCa2 cells with the HDAC inhibitor trichostatin A led to ~2.5-fold upregulation of Dab2 mRNA levels; however, cotreatment with both inhibitors failed to induce an additive effect. Similarly, E-cadherin mRNA levels were significantly induced by 5-azacytidine treatment as compared to trichostatin A treatment, suggesting that promoter methylation may be the primary mechanism for downregulation of both Dab2 and E-cadherin expression in MiaPaCa2 cells. While 5-azacytidine inhibits DNA methylase activity, alteration of other cellular pathways may occur, such as cell cycle inhibition, which may indirectly alter Dab2 levels. No evidence of promoter methylation was found in normal pancreatic tissue samples; however, Dab2 promoter methylation was observed in 3/5 Stage I pancreatic cancer tumor samples (Fig. 6c). In addition, Dab2 promoter methylation was observed in 1/5 of metastatic pancreatic tumor samples, while no Dab2 promoter methylation was seen in Stage II or Stage III tumor samples (Fig. 6c). These results suggest that the decreased Dab2 expression observed in Stage I tumors (Fig. 1a) may be in part due to promoter methylation of Dab2.

## Discussion

Pancreatic cancer is a highly lethal disease, which has been linked to early dissemination of metastatic cells and enhanced chemoresistance; characteristics of pancreatic cancer cells which have undergone EMT^4^. One of the key hallmarks of cells which have undergone EMT is loss of expression of the adhesion molecule E-cadherin. In a study of resected human pancreatic tumors, it was found that appearance of EMT was associated with poor survival^8^, and low E-cadherin expression was found to be associated with dedifferentiation, invasion, and a high incidence of lymph node metastasis^9–11^. In the current study, a significant positive correlation between expression of Dab2 and E-cadherin mRNA was observed in pancreatic cancer cell lines, while downregulation of Dab2 expression in the Panc1 cell line was shown to lead to a significant decrease in E-cadherin mRNA and protein expression, suggesting that Dab2 directly or indirectly controls E-cadherin expression. The consequence of decreased E-cadherin expression early in pancreatic cancer progression has recently been demonstrated in a mouse model of pancreatic cancer^46^. Orthotopic injection of E-cadherin (−) tumor-initiating cells isolated from early stage PanIN mice gave rise to tumors with a shorter latency than E-cadherin (+) cells; however, E-cadherin expression status failed to elicit a significant effect on latency using tumor-initiating cells isolated from late stage PDAC mice^46^. In the current study, both Dab2 and E-cadherin mRNA levels were significantly decreased as early as Stage I pancreatic tumors (Fig. 5), suggesting that loss of Dab2 early in pancreatic progression may lead to decreased E-cadherin expression, thereby contributing to the early dissemination of pancreatic cancer. In support of this hypothesis, interrogation of the pancreatic adenocarcinoma TCGA cancer genomic dataset identified that Dab2 mRNA levels were higher in patient tumor samples designated as disease-free versus those whose disease had recurred or progressed (Fig. 5c,d).

While TGFβ has been shown to act as a potent tumor suppressor in normal epithelial cells, it also acts as a pro-metastatic factor late in tumor progression, a finding that has been attributed, in part, to its ability to stimulate EMT and the CSC phenotype^47,48^. We have previously shown that loss of Dab2 expression in normal mammary epithelial cells leads to increased expression of TGFβ2 and a constitutive EMT phenotype^28^, and in squamous cell carcinoma (SCC), low Dab2 expression in combination with high TGFβ2 expression in tumors was correlated with reduced survival^27^. In this study, decreased expression of Dab2 in COLO357 and Panc1 pancreatic cancer cell lines altered the expression of genes involved in the EMT/CSC phenotype mediated by TGFβ (Figs 2, 3). While treatment with the selective TGFβR/RII inhibitor LY2109761 did not alter mRNA expression for all interrogated EMT/CSC marker genes, mRNA expression of E-cadherin was significantly increased and CD133 was significantly decreased in the Panc1 1059 and COLO357 1059 cell lines, respectively (Fig. S1a,b), suggesting that Dab2 indirectly affects EMT, in part, through modulation of the TGFβ signaling pathway.

Activation of MAPK activity has been postulated to synergize with TGFβ in induction of EMT^38^, while maintenance of EMT through establishment of an autocrine TGFβ signaling loop has been shown to require high levels of MAPK activity^49^. The appearance of the EMT phenotype, along with activated MAPK signaling, was found to confer a poor prognosis in surgically resected pancreatic cancer^8^. Mutations in the *KRas* gene, which are observed in 90% of PDAC cases, result in generation of a constitutively active form of the protein leading to downstream activation of the MAPK/ERK signaling pathway. We have previously shown that loss of Dab2 leads to increased Ras activation in mammary epithelial cells, upregulation of TGFβ2 levels, and constitutive EMT, which could be abrogated by treatment with MAPK inhibitors^28^. In this study, loss of Dab2 expression in the COLO357 cell line, a cell line that expresses wild-type K-Ras protein, led to increased p-ERK1/2 levels, indicating activation of the MAPK signaling pathway (Fig. 2a). In addition, loss of Dab2 in COLO357 cells led to activation of TGFβ signaling evidenced by increased expression of CD133, which could be decreased by treatment with the selective TβRI/RII inhibitor LY2109761 (Figs 3 and S1b). Loss of Dab2 in the Panc1 1059 cell line did not alter p-ERK1/2 levels, as the Panc1 cell line harbors mutant K-Ras; however, loss of Dab2 expression led to activation of the TGFβ signaling pathway, as evidenced by the reversal in E-cadherin downregulation and vimentin upregulation with LY2109761 treatment (Fig. S1a). Further, Dab2 overexpression in the Panc1Dab2 cell line blocked TGFβ-stimulated EMT (Fig. 4), suggesting that Dab2 may interrupt the crosstalk between the two pathways. In pancreatic tumors or cell lines which harbor oncogenic K-Ras, such as the Panc1 cell line, downregulation of Dab2 expression would provide the activation of the TGFβ signaling pathway. In the case of tumors or cell lines which harbor wild-type K-Ras, such as the COLO357 cell line, loss of Dab2 expression would lead to activation of both the MAPK and TGFβ pathways. Together, these results suggest that loss of Dab2 in pancreatic cancer may lead to cooperation between the MAPK and TGFβ signaling pathways required for stable EMT, regardless of the mutational status of K-Ras.

Results presented in this study show that Dab2 may act as a tumor suppressor in pancreatic cancer by inhibition of TGFβ-stimulated EMT and the CSC phenotype. However, Dab2 may play an indirect role as a tumor suppressor in pancreatic cancer by affecting TGFβ levels within the tumor microenvironment through its modulation of the cell-surface expression of the TGFβ receptors. Dab2 has previously been shown to facilitate TGFβ receptor trafficking from early endosomes to recycling endosomes, where loss of Dab2 expression resulted in impaired endosomal trafficking and loss TGFβ receptor recycling to the cell surface^20^. Restoration of Dab2 expression in SK-BR-3 breast cancer cells was shown to promote TGFβ depletion from the surrounding media through receptor trafficking; and these decreased TGFβ levels inhibited production of regulatory T cells (Tregs) from naïve T cells^50^. Both increased levels of TGFβ ligand and tumor-infiltrating Tregs have been associated with poor prognosis in pancreatic cancer^15,51^. Whether the primary role of Dab2 as a tumor suppressor in pancreatic cancer is through a direct effect on the tumor cells themselves or through effects on the tumor microenvironment remains to be determined.

Methylation of the Dab2 promoter has previously been found to occur frequently in SCC^27^, lung^29^, nasopharyngeal^30^ and hepatocellular carcinoma^31^, while decreased Dab2 levels observed in breast cancer are not due to promoter methylation^23^. Dab2 promoter methylation in this study was found in 3/5 Stage I tumors, while promoter methylation was absent in Stage II and Stage III tumors (Fig. 6). These results indicate that Dab2 promoter methylation may play a more prominent role in modulation of Dab2 expression early in pancreatic cancer progression while other mechanisms for decreased Dab2 expression may predominate in later stages of pancreatic cancer. In addition, Stage I and metastatic lesions may have a higher percentage of CSC populations such that while the methylation may persist through all stages, methylation in Stage II and Stage III tumors cannot be detected by the employed methodology. Determination of the mechanism for decreased expression of Dab2 in pancreatic cancer and whether methylation is a significant factor requires further analysis in additional tumor samples.

In summary, these results show that decreased Dab2 mRNA levels are observed in pancreatic cancer as compared to normal pancreatic tissue and may serve as a prognostic marker for cancer progression. In addition, we find that loss of Dab2 expression triggers the appearance of the EMT/CSC phenotype which can in part be attributed to modulation of the TGFβ signaling pathway. As activation of the TGFβ signaling pathway has been linked to metastasis, chemoresistance, and poor prognosis, a further understanding of the role and regulation of Dab2 during pancreatic cancer progression may identify novel targets for therapeutic intervention.

## Methods

### Pancreatic tumor and tissue samples

The de-identified pancreatic tumor and normal tissue samples used in this study were obtained from the Indiana University Simon Cancer Center Tissue Procurement & Distribution Core. All the tumor samples were histologically confirmed as pancreatic ductal adenocarcinoma and TNM stage was provided (Table S2). Normal pancreatic tissue samples used in the study were designated as normal adjacent tissue obtained from various pancreatic pathologies (Table S2). This study was approved by the Indiana University Institutional Review Board (exempt IRB #NS1002-06) in accordance with approved guidelines. The samples consisted of: 5 histologically confirmed normal pancreatic tissue samples, 5 Stage I, 5 Stage II, 3 Stage III and 5 metastatic tumor samples. The cBioPortal website (https://cBioPortal.org) was used to obtain mRNA levels of Dab2 in PDAC, stratified by disease-free and recurred/progressed status. The results shown are based on data generated by the TCGA Research Network: http://cancergenome.nih.gov/. Dab2 mRNA expression for individual groups is shown in Table S3.

### Cell lines and culture conditions

AsPC1, BxPC3, Panc1 and MiaPaCa2 cell lines were obtained from ATCC while the COLO357 cell line was obtained from Dr. Scott Kern^34^. Parental cell lines were cultured in either DMEM or RPMI1640 media (Sigma-Aldrich) supplemented with 10% FBS (Atlanta Biologicals) and 1% antibiotic/antimycotic solution (Gemini Systems) at 37 °C and 5% CO_2_. Stable ablation of Dab2 in COLO357 and Panc1 cells by shRNA-mediated technology was achieved using the pSIREN-RetroQ vector system (Clontech) as previously described^28^. For overexpression studies, wild-type Dab2^21^ was cloned into pBabepuro (Addgene) and verified by sequencing. Retrovirus containing the targeted sequences were generated by co-transfecting HEK293T cells with the either the pSIREN or pBabepuro vectors and the pCL10A1 packaging plasmid. Retrovirus was added to cells (in the presence of 8 μg/ml polybrene) for 4hrs, followed by addition of standard media. After an additional 24 hrs, cells were selected in media containing 1 mg/ml puromycin, and maintained as stable pools of clones in the same media. Treatment of cells to stimulate EMT was performed by the addition of 2.5 ng/mL TGFβ1 (R & D Systems) for 72 hours. For blockade of TGFβ signaling, cells were treated with with LY2109761 (Cayman Chemical) for 48 hrs prior to isolation of mRNA. For DNMT inhibition studies, MiaPaCa2 cells were treated with 5 μM 5-aza-2′deoxycytidine (Sigma) for 3 days, with media refreshed each day. For HDAC inhibition studies, MiaPaCa2 cells were treated for 1 day with 100 ng/mL trichostatin A (Sigma) prior to mRNA isolation. For concurrent treatment, trichostatin A was added to the cell culture for the last 24 hrs of 5-aza-2′deoxycytidine treatment.

### Quantitative RT-PCR analysis

Total RNA was isolated from cell lines (RNeasy, Qiagen) and from pancreatic tissue or tumor samples (Trizol, Invitrogen) per manufacturer’s instructions. 2.5–5.0 µg of total RNA was reverse-transcribed (RT) with Superscript II reverse transcriptase (Invitrogen) using random hexamers (Roche) for priming. Real-time PCR was performed using FastStart Universal SYBR Green Master Mix (Roche) and gene-specific primers on an Illumina Eco Real-time PCR System. Primer pairs for specific genes were designed using the Primer Express program (Applied Biosystems), with cyclophilin amplification used as the endogenous control (Table S4). Samples were measured in triplicate and analyzed by the threshold cycle (Ct) comparative method. The 2^−ΔΔCt^ value was calculated, where ΔCt = Ct_target_ − Ct_cyclophilin_, and ΔΔCt = ΔCt_sample_ − ΔCt_reference_. Relative quantitation for each gene is shown ± standard deviation.

### Western analysis

Cells were lysed in Buffer D (20 mM Tris, pH 7.5; 137 mM NaCl; 2 mM EDTA; 1% Triton X-100, 10% glycerol, COMPLETE protease inhibitor and PhosSTOP phosphatase inhibitor, Roche). Equal amounts of protein were resolved on SDS/PAGE gels, transferred to PVDF membrane (Immobilon, Millipore) and subjected to Western analysis. Immunoblots were visualized utilizing enhanced chemiluminescence (ECL-Plus, GE Healthcare) and autoradiography film. Quantitation of protein expression was performed using ImageJ analysis software. The following antibodies were used for Western analysis: Dab-2/p96 (610465), Ecadherin (610181), Ncadherin (610921) from BD Transduction Laboratories; p-ERK1/2 (sc-7383), ERK2 (sc-154), HSP90 α (sc-7947), and Vimentin (sc-5565) from Santa Cruz Biotechnology.

### Flow cytometry

Cells for flow cytometry were harvested with 0.05% trypsin/0.025% EDTA to generate single cell suspensions. Cells were washed and resuspended in wash buffer (PBS with 0.1% BSA) at a concentration of 2 × 10^6^ cells/ml. Cells (1 × 10^6^) were incubated with the specified fluorochrome-conjugated antibodies (1 μg) or their respective isotype controls for 30 min at 4 °C in the dark. Cells were washed twice with wash buffer, resuspended in 0.5 ml of wash buffer containing 0.2 μg/ml DAPI for gating of live cells and analyzed on a LSRII flow cytometer (Becton Dickinson) using FACSDiva software. Antibodies used were: BD Biosciences: Anti-EpCAM PerCP-Cy5.5 (BD 347199), Mouse IgG1 PerCP-Cy5.5 isotype control (347212), PE Anti-human CD24 (BD 555428), PE Mouse IgG1 isotype control (BD555749), FITC Anti-human Cd44 (BD 555478) FITC Mouse IgG2a isotype control; and eBioscience: APC-Anti CD133 (171338-42) and FITC and APC Mouse IgG1 isotype control (22-1714-79).

### Methylation-specific PCR

Genomic DNA was isolated from cell lines (DNeasy, Qiagen) and from pancreatic tissue or tumor samples (Trizol, Invitrogen) per manufacturer’s instructions. DNA was subjected to bisulfite conversion using the EZ DNA Methlyation-Gold Kit (Zymo Research) according to manufacturer’s instructions. Methylation of exon1 of the Dab2 gene was determined using bisulfite-modified DNA (1 μg) using primers specific for either the unmethylated or methylated sequences as in Bagadi *et al*.^23^. Briefly, samples were subjected to denaturation at 95 °C for 5 min, followed by 35 cycles of 95 °C for 30 sec, 60 °C for 30 sec, and 72 °C for 1 min with a final incubation at 72 °C for 5 min. Samples were separated on a 2% agarose gel and visualized using ethidium bromide. Primers utilized were the following: Dab2 U Forward 5′-GAATTATATTTTTTGTTGGGAGTGGTTGT-3′; Dab2 U Reverse 5′-CCAACTAACTATTACCTCCATAAAACA-3′; Dab2 M Forward 5′-TATTTTTCGTCGGGAGTGGTCGC-3′; Dab2 M Reverse 5′-ACTAACTATTACCTCCGTAAAACG-3′.

### Statistical analysis

Statistical comparisons were performed using One-way ANOVA followed by Tukey’s post-hoc test where appropriate. *P* values less than 0.05 were considered significant.

## Supplementary information

Supplemental Data
