# Supplementary material for: Loss of Disabled-2 Expression in Pancreatic Cancer Progression
Source: Sci Rep. 2019 May 17;9:7532. doi: 10.1038/s41598-019-43992-z (PMC6525241; doi:10.1038/s41598-019-43992-z)
Supplement: Supplementary file 1 — Supplemental Data [file 41598_2019_43992_MOESM1_ESM.docx]

**Supplemental Data:**

**Loss of Disabled-2 Expression in Pancreatic Cancer Progression**

Barbara A. Hocevar

**Supplementary Table S1: Characterization of gene expression in pancreatic cell lines***

| ***Cell line*** | ***Differentiation*** | ***K-Ras*** | ***p53*** | ***Smad4*** | ***Dab2*** | |
| --- | --- | --- | --- | --- | --- | --- |
|  |  |  |  |  | ***mRNA*** | ***Protein*** |
| *COLO 357* | *Moderate* | *Wt* | *Wt* | *HD* | *++++* | *++++* |
| *AsPC1* | *Poor* | *Mut* | *Mut* | *WT/HD/Mut^#^* | *++* | *+* |
| *BxPC3* | *Moderate* | *Wt* | *Mut* | *HD* | *++* | *++* |
| *PANC-1* | *Poor* | *Mut* | *Mut* | *Wt* | *+* | *+* |
| *MiaPaCa2* | *Poor* | *Mut* | *Mut* | *Wt* | *-/+* | - |

*Wt, Wild-type; Mut, Mutant; HD, Homozygous deletion

#Conflicting reports on status

| **Stage** | **Specific Site** | **Histologic Type** | **Met Site** | **T** | **N** | **M** | **Comments** |
| --- | --- | --- | --- | --- | --- | --- | --- |
| **NL** | NORMAL TISSUE | ADENOCARCINOMA |  |  |  |  |  |
|  | NORMAL TISSUE | ADENOCARCINOMA |  |  |  |  |  |
|  | NORMAL TISSUE | ISLET CELL CARCINOMA |  |  |  |  |  |
|  | NORMAL TISSUE | ADENOCARCINOMA |  |  |  |  |  |
|  | NORMAL TISSUE | IPMN (borderline malignant) |  |  |  |  |  |
| **I** | PANCREAS, NOS | ADENOCARCINOMA, NOS |  | T1 | N0 | Mx |  |
|  | PANCREAS, NOS | ADENOCARCINOMA, NOS |  | T1 | N1 | Mx |  |
|  | PANCREAS, NOS | ADENOCARCINOMA, NOS |  | T1 | N1 | Mx |  |
|  | HEAD OF PANCREAS | ADENOCARCINOMA, NOS |  | T1 | N0 | Mx |  |
|  | PANCREAS, NOS | ADENOCARCINOMA, NOS |  | T1 | N0 | Mx |  |
| **II** | HEAD OF PANCREAS | ADENOCARCINOMA, NOS |  | T3 | N1 | Mx |  |
|  | PANCREAS, NOS | ADENOCARCINOMA, NOS |  | T3 | N1 | Mx |  |
|  | PANCREAS, NOS | ADENOCARCINOMA, NOS |  | T3 | N1 | Mx |  |
|  | PANCREAS, NOS | ADENOCARCINOMA, NOS |  | T3 | N1 | Mx |  |
|  | PANCREAS, NOS | ADENOCARCINOMA, NOS |  | T3 | N1 | Mx |  |
| **III** | PANCREAS, NOS | ADENOCARCINOMA, NOS |  | T4 | N1 | Mx |  |
|  | PANCREAS, NOS | ADENOCARCINOMA, NOS |  | T4 | N1 | Mx | 80%T; 20% stroma |
|  | PANCREAS, NOS | ADENOCARCINOMA, NOS |  | T4 | N1 | Mx | 70%T,30% stroma |
| **IV** | HEAD OF PANCREAS | ADENOCARCINOMA, NOS | TORSO-ABDOMEN |  |  |  |  |
|  | HEAD OF PANCREAS | EMBRYONAL CARCINOMA, NOS | GI-LIVER |  |  |  |  |
|  | BODY OF PANCREAS | NEUROENDOCRINE CARCINOMA | GI-LIVER |  |  |  | 90%T,10%NEC |
|  | PANCREAS, NOS | CARCINOMA, NOS | TORSO-GROIN/  PELVIS |  |  |  | 90%T,10%NEC |
|  | AMPULLA OF VATER | CARCINOMA, NOS | TORSO-ABDOMEN |  |  |  |  |

**Supplementary Table S2: Characteristics of Pancreatic Tissue Samples**

|  |  | **DAB2 mRNA**  **(ave ± SD)** | **n** | **p*** |
| --- | --- | --- | --- | --- |
| **Stage I** | Disease Free | 1559.84 ± 1165.39 | 11 |  |
|  | Recurred/Progressed | 1864.30 ± 781.89 | 7 | 0.55 |
| **Stage II** | Disease Free | 2354.79 ± 1318.74 | 39 |  |
|  | Recurred/Progressed | 1915.15 ± 790.76 | 71 | 0.04 |
| **Stage III + IV** | Disease Free | 2696.01 ± 318.28 | 2 |  |
|  | Recurred/Progressed | 1584.46 ± 930.38 | 4 | 0.19 |
| **All** | Disease Free | 2199.75 ± 1296.31 | 52 |  |
|  | Recurred/Progressed | 1894.68 ± 789.26 | 82 | 0.09 |
|  |  |  |  |  |
| **Grade 1** | Disease Free | 1330.53 ± 783.90 | 15 |  |
|  | Recurred/Progressed | 2114.11 ± 730.44 | 11 | 0.02 |
| **Grade 2** | Disease Free | 2533.23 ± 1008.10 | 30 |  |
|  | Recurred/Progressed | 1713.93 ± 646.60 | 42 | 0.0001 |
| **Grade 3 + 4** | Disease Free | 2263.34 ± 2062.70 | 10 |  |
|  | Recurred/Progressed | 2143.21 ± 885.59 | 28 | 0.80 |
| **All** | Disease Free | 2156.15 ± 1293.70 | 55 |  |
|  | Recurred/Progressed | 1916.67 ± 768.48 | 81 | 0.0004 |
| # retrieved from cBioPortal-TCGA database  * p-value calculated between disease-free and recurred/progressed | | | | |

**Supplementary Table S3: DAB2 mRNA levels in PDAC#**

**Supplementary Table S4.** Oligonucleotide primers used for qRT-PCR analysis

| **Gene Name** | **Symbol** | **Forward Primer 5’→3’** | **Reverse Primer 5’→3’** |
| --- | --- | --- | --- |
| Cyclophilin | PPIH | ATGGTCAACCCCACCGTGT | TCTGCTGTCTTTGGGACCTTGT |
| Bmi-1 | BMI1 | GCCAACAGCCCAGCAGGAGG | TTGGTGGTTACCGCTGGGGC |
| E-Cadherin | CDH1 | TGCCCAGAAAATGAAAAAGG | GTGTATGTGGCAATGCGTTC |
| N-Cadherin | CDH2 | ACAGTGGCCACCTACAAAGG | CCGAGATGGGGTTGATAATG |
| Disabled-2 | DAB2 | GCTGTGTCCACTCAGCCCCC | GGTGGCTGCCGCAGTTGGAA |
| Nanog | NANOG | ACCTGGTGCACCCAATCCTGG | GGCCTTCCCCAGCAGCTTCC |
| Oct4 | POU5F1 | AAAGGGTGGGGGCAGGGGAG | TGTGTCTATCTACTGTGTCCCAGGC |
| Snail | SNAI1 | ATGCCGCGCTCTTTCCTCGTC | TCAGCGGGGACATCCTGAGCAG |
| Slug | SNAI2 | CTTTTTCTTGCCCTCACTGC | ACAGCAGCCAGATTCCTCAT |
| Sox2 | SOX2 | AGAACCCCAAGATGCACAAC | CGGGGCCGGTATTTATAATC |
| Vimentin | VIM | GAGAACTTTGCCGTTGAAGC | GCTTCCTGTAGGTGGCAATC |
| Zeb1 | ZEB1 | TGCACTGAGTGTGGAAAAGC | TGGTGATGCTGAAAGAGACG |
| Zeb2 | ZEB2 | CGCTTGACATCACTGAAGGA | CTTGCCACACTCTGTGCATT |

**Supplementary Figure S1: Inhibition of the TGFβ signaling pathway in Dab2 KD cells partially reverts EMT and CSC gene expression patterns**

**a**

**b**

**Supplementary Figure S2: Western blots cropped for Figure 1a*****

**(1a1) Western blot: α-Dab2**


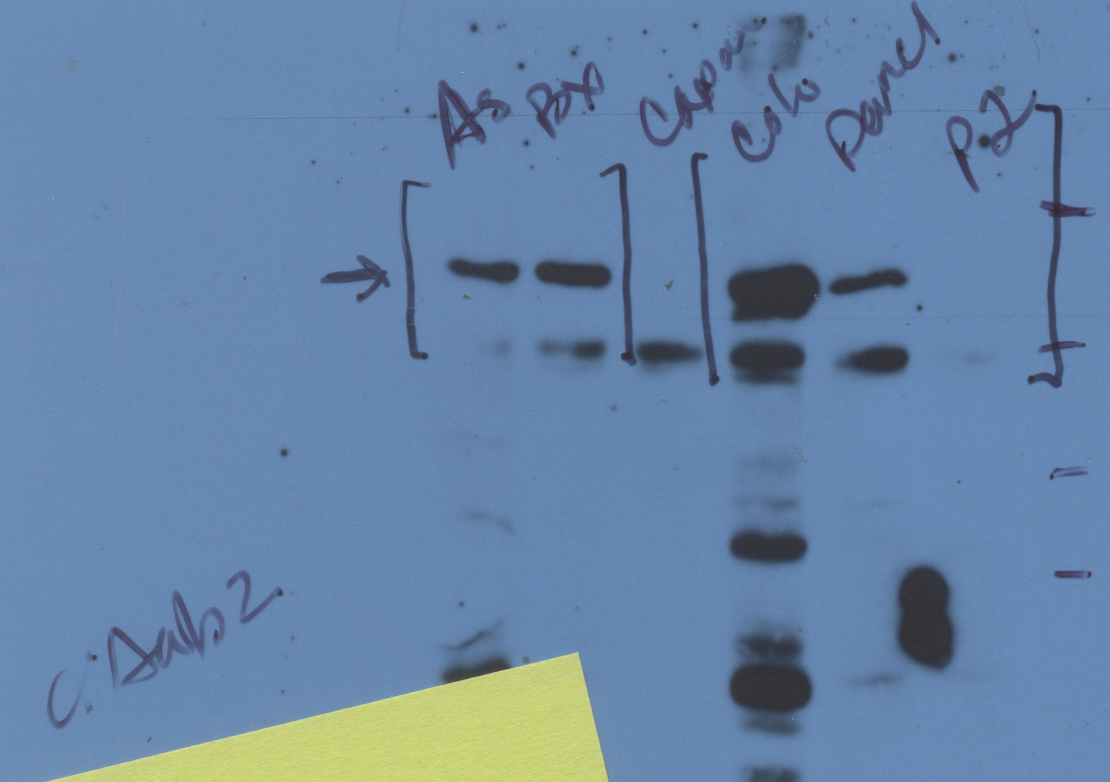


**(1a2) Western blot: α-Ncadherin**


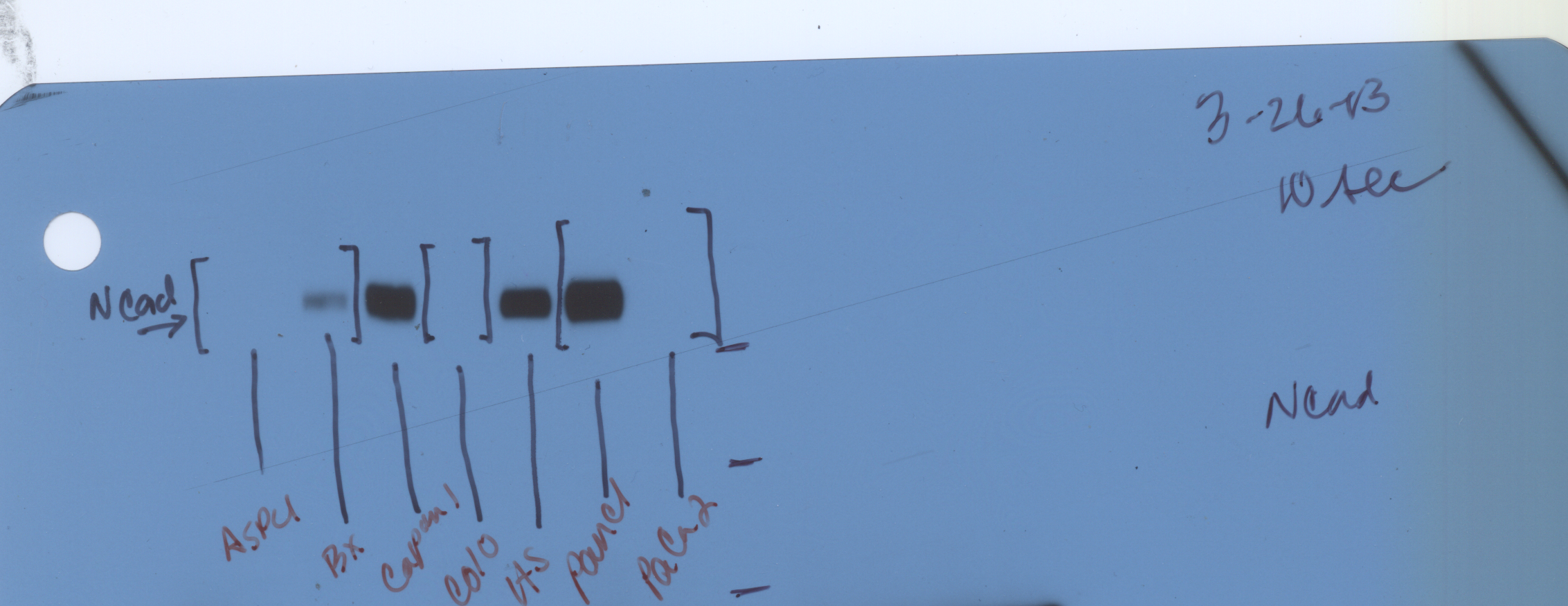


**(1a3) Western blot: α-vimentin**


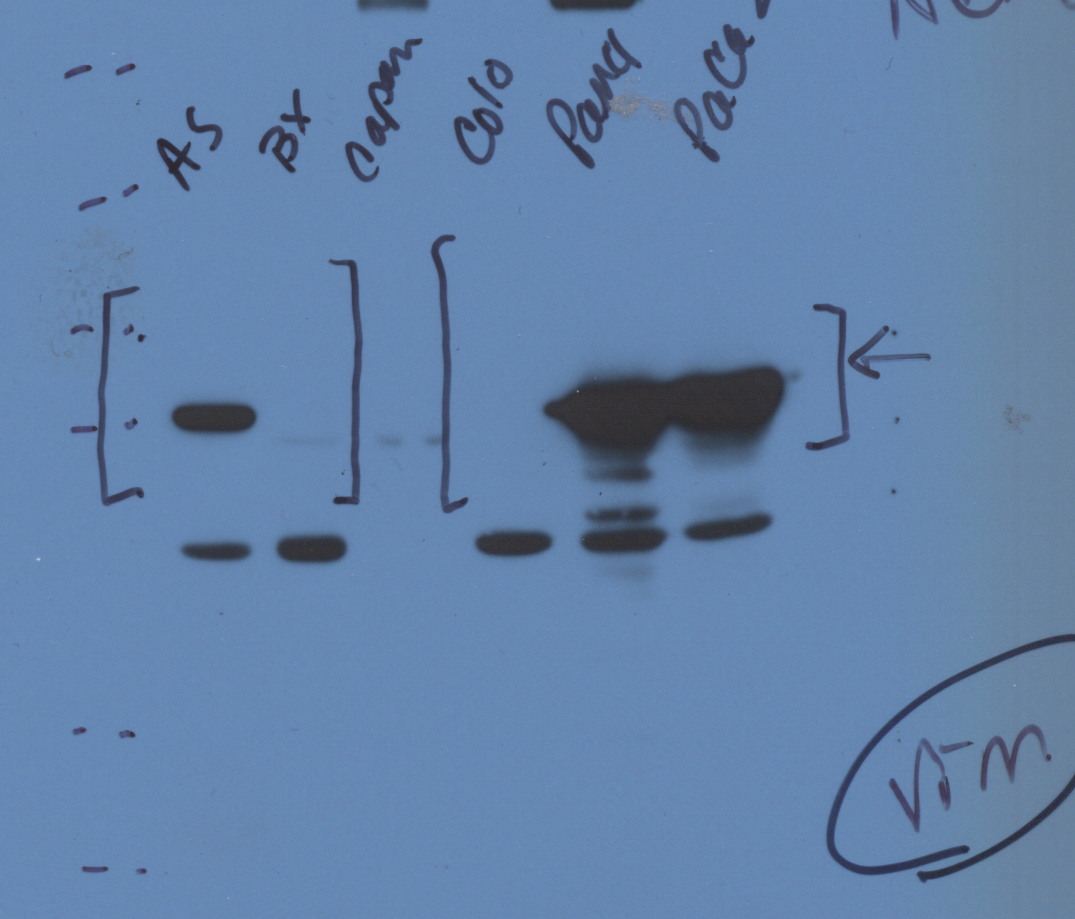


**(1a4) Western blot: α-HSP90**

**
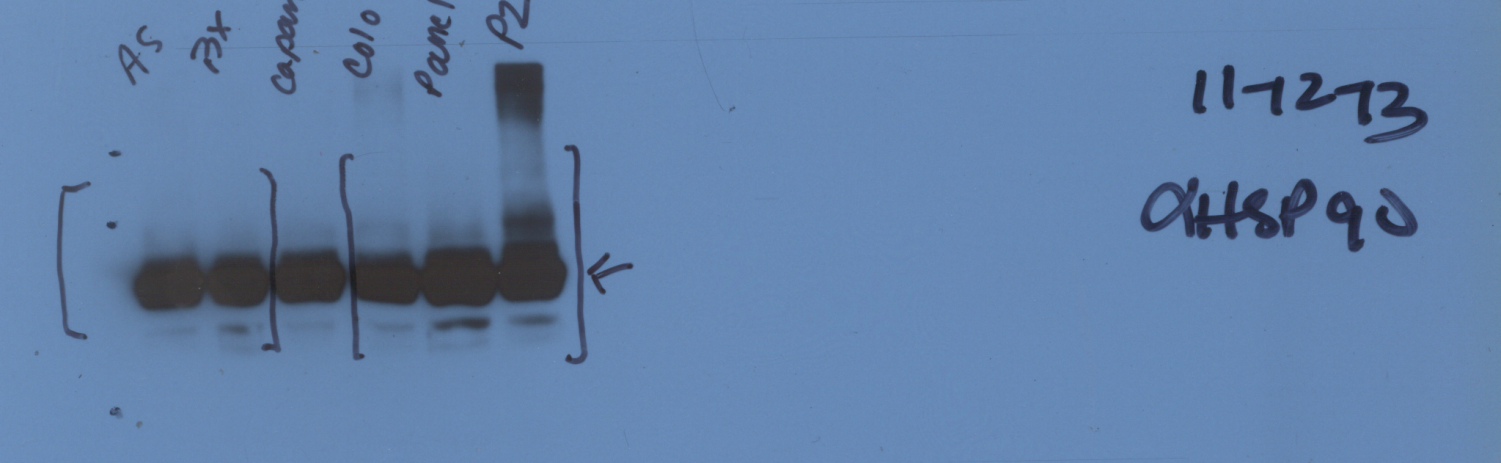
**

**(1b1) Western blot: α-Ecadherin**


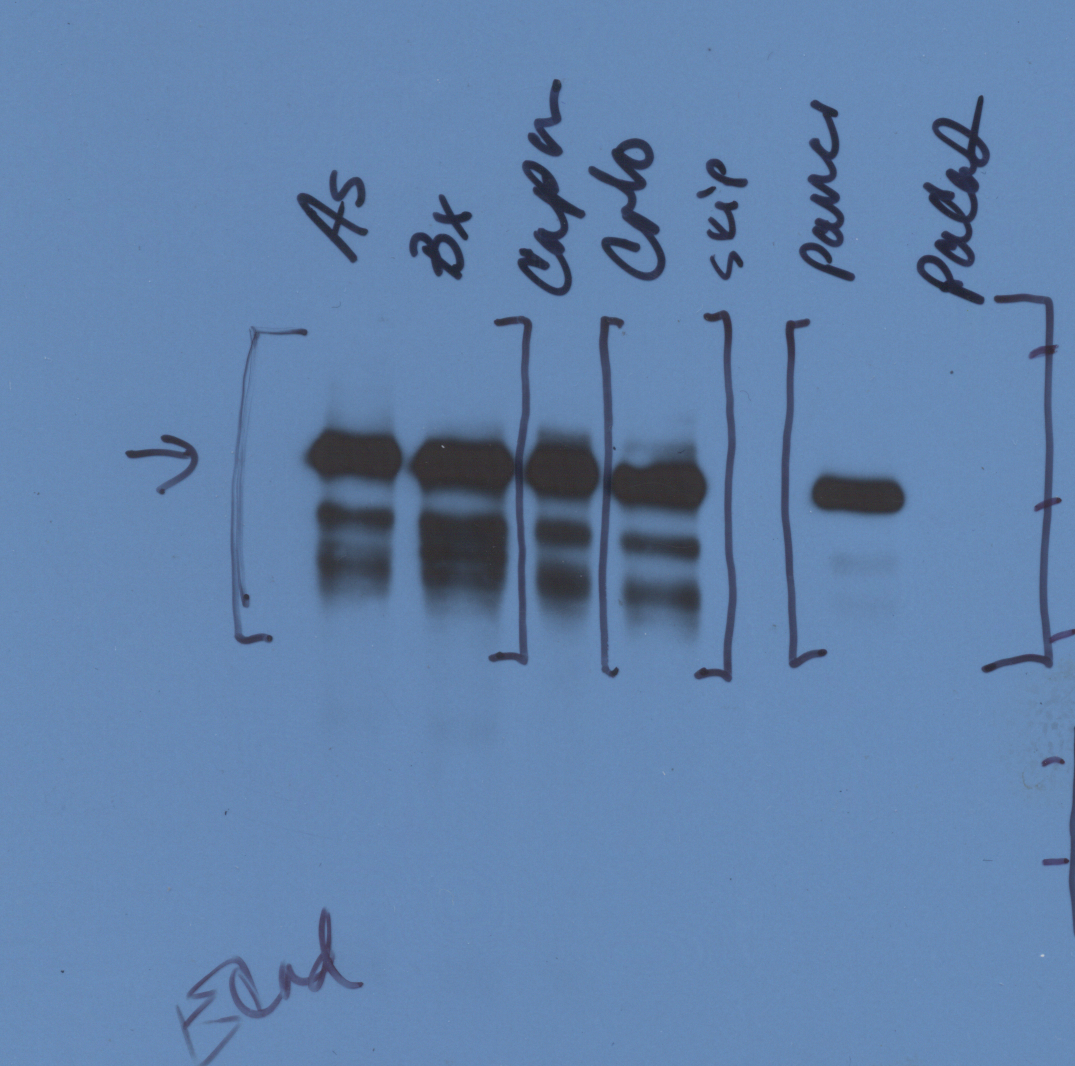


*****Note: Western blots for all proteins were performed on the same samples, but not at the same time. Only AsPC1, BxPC3, COLO357, Panc1 and MiaPaCa2 cell lines were included in this study. Bands in brackets were used to generate Fig 1; arrow indicates protein of interest.**

**Supplementary Figure S3: Western blots cropped for Figure 2a (4) and 2b (2)**

**(2a1) Western: α-Dab2**

**

**

**(2a2) Western: α-phosphoERK**

**
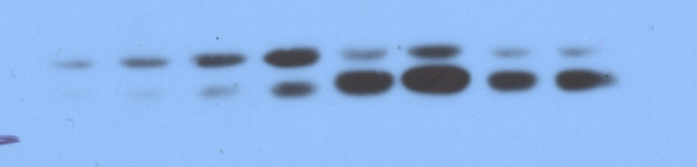
**

**(2a3) Western: α-total ERK**

**
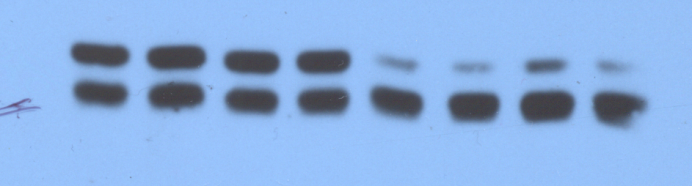
**

**(2a4) Western: α-HSP90**

**
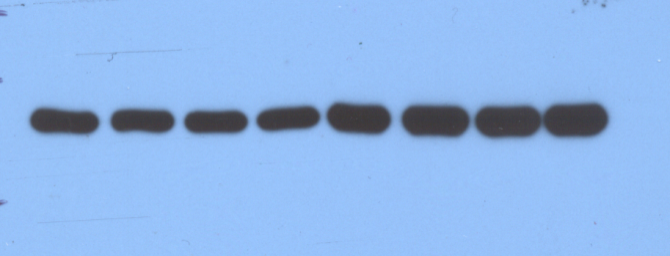
**

**(2b1)Western: α-Ecadherin**

**
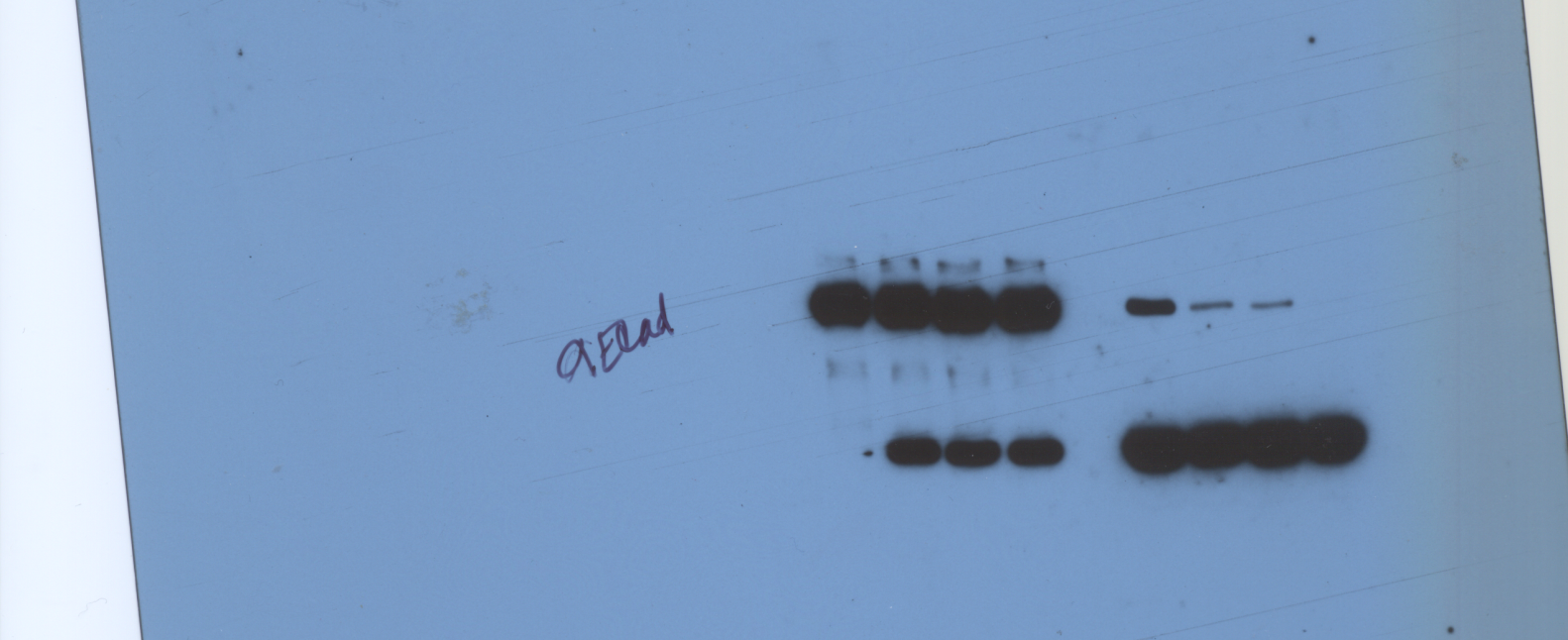
**

**(2b2)Western: α-HSP90**

**

**

**Supplementary Figure S4: Western blots cropped for Figure 4a (3)**

**(4a1) Western: α-Dab2**

**
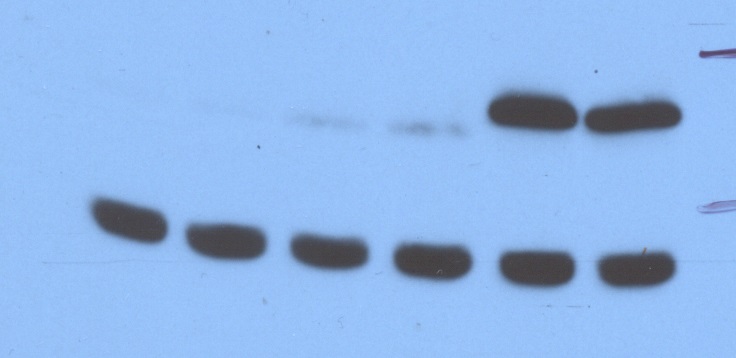
**

**(4a2) Western: α-Ecadherin**

**
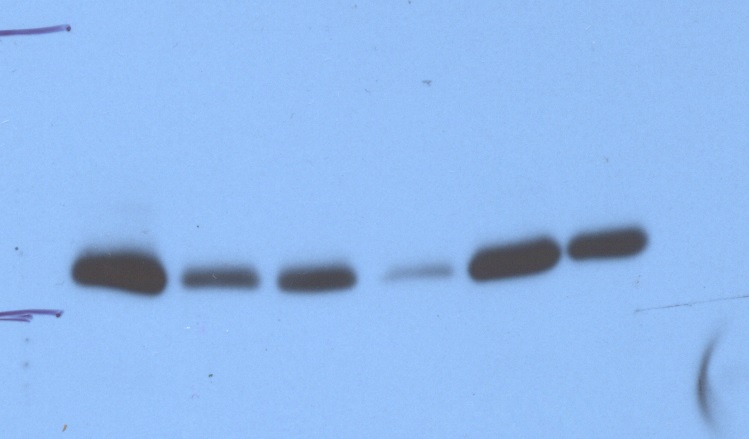
**

**(4a3) Western: α-HSP90**

**
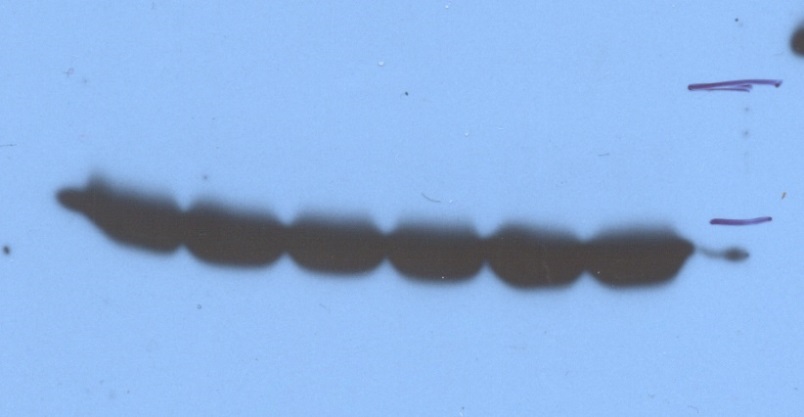
**
